# The crucial role of thromboxane A_2_ in colorectal cancer development and metastasis: a key target for anticancer therapy

**DOI:** 10.3389/fphar.2026.1787936

**Published:** 2026-04-30

**Authors:** Sara Di Berardino, Stefania Tacconelli, Annalisa Contursi, Huzaifa Ali Ahfaz, Paola Patrignani

**Affiliations:** Systems Pharmacology and Translational Therapeutics Laboratory, at the Center for Advanced Studies and Technology (CAST), and Department of Neuroscience, Imaging and Clinical Science, “G. d'Annunzio” University Medical School, Chieti, Italy

**Keywords:** aspirin, colorectal cancer, immunity, platelets, TXA_2_, TXA_2_ receptors

## Abstract

Platelets play a crucial role in atherothrombosis, as demonstrated by the effectiveness of the antiplatelet agent aspirin (acetylsalicylic acid, ASA) in the secondary prevention of cardiovascular disease. Low-dose aspirin (75–100 mg/day) irreversibly and selectively inhibits cyclooxygenase (COX)-1 activity in platelets, thereby reducing thromboxane (TX)A_2_-dependent platelet activation. Recent evidence suggests that persistent platelet activation contributes to early carcinogenesis and metastasis progression by releasing TXA_2_, which acts through two distinct mechanisms. First, by promoting inflammation at gastrointestinal mucosal lesions via induction of COX-2 and increased prostaglandin E_2_ biosynthesis; second, by curbing T-cell-mediated anticancer immune responses. Therefore, inhibition of platelet TXA_2_ biosynthesis is considered a central mechanism of aspirin’s anticancer effects. Recent findings from large randomized clinical trials support its use in preventing Lynch syndrome cancers and as an adjuvant treatment in colorectal cancer patients with tumors carrying PI3K pathway mutations. However, it is important to note that aspirin and other conventional antiplatelet agents may increase the risk of bleeding. Novel antiplatelet agents are currently under development to achieve effective antiplatelet activity while minimizing bleeding. This review reports three possible options: PIM kinase inhibitors, protein disulfide isomerase inhibitors, and 12-lipoxygenase inhibitors. In conclusion, the discovery of novel roles for platelet TXA_2_ in cancer paves the way for new therapeutic uses of low-dose aspirin and potentially other antiplatelet agents. Further research is needed to elucidate the mechanisms underlying aspirin’s anticancer effects fully and to advance personalized treatment strategies. Furthermore, developing safer antiplatelet drugs will lead to therapeutic approaches for the primary prevention of cardiovascular disease and cancer.

## 1 Introduction

**FIGURE 1:**
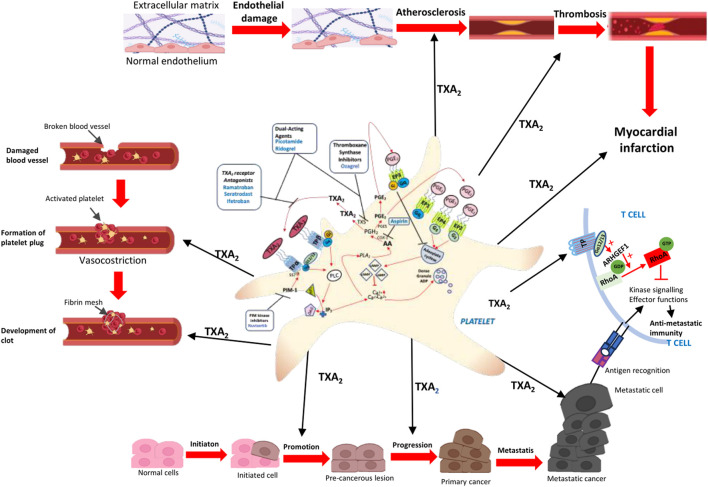
The multifunctional platelet. Following vascular wall injury, platelets undergo a sequence of functional responses, including adhesion, spreading, granule secretion, aggregation, exposure of a procoagulant surface, microparticle formation, and clot retraction (Harrison, 2005). Collectively, these events lead to the rapid formation of a haemostatic plug that occludes the site of injury and prevents blood loss (Rodgers, 1999). Alterations in one or more of these functions and/or in platelet number generally result in impaired haemostasis, with a consequent increased risk of bleeding. In contrast, a marked increase in platelet number or reactivity may promote inappropriate thrombus formation that may also develop within atherosclerotic plaques, leading to stroke and myocardial infarction, which represent two of the leading causes of cardiovascular morbidity and mortality (Michelson, 2002). Platelets also play a key role in tumor progression and metastasis. After tumor cells enter the bloodstream, they rapidly activate platelets, which form a protective coating that shields them from hemodynamic shear forces and the cytotoxic activity of NK cells (Michelson, 2002; Gay and Felding-Habermann, 2011). Once activated, platelets release a plethora of lipid mediators, including TXA_2_, ADP, and proteins, such as growth and angiogenic factors. Additionally, activated platelets shed extracellular vesicles that deliver several factors, including RNA, microRNAs, and proteins, to other cells. The activation of specific platelet receptors, such as those involved in TXA_2_ signaling pathways, contributes to tumor cell survival, proliferation, and metastatic potential (Nie et al., 2000). In addition, activated platelets contribute to metastatic progression by modulating the antitumor immune response. In particular, TXA_2_ released by activated platelets binds to the TP receptors on T cells, thereby triggering an immunosuppressive signaling cascade. This interaction activates ARHGEF1, a guanine nucleotide exchange factor that converts RhoA from its inactive GDP-bound form to its active GTP-bound state (Yang et al., 2025). Activation of RhoA inhibits kinase signaling pathways downstream of the T cell receptor (TCR), resulting in reduced T cell proliferation and effector functions and suppression of anti-metastatic immunity (Yang et al., 2025). Through this mechanism, platelets promote the survival of circulating tumor cells and facilitate their colonization of distant organs. Because TXA_2_ production by platelets depends on COX-1 activity, inhibition of this pathway by aspirin or selective COX-1 inhibitors effectively restores T cell–mediated antitumor immunity and counteracts metastatic progression (Yang et al., 2025).

Platelets are small anucleate cells derived from megakaryocytes in the bone marrow or lung (Lefrançais et al., 2017). Their lifespan is limited to 5–7 days in humans (Holinstat, 2017). Platelets are multifunctional cells involved in hemostasis and thrombosis, as well as in vascular repair, inflammation, atherosclerosis, and tumor metastasis (Harrison, 2005). Under inflammatory conditions, platelets can extravasate into tissues, contributing to microvascular dysfunction and immune recruitment (De La Cruz et al., 2021). Upon activation, they release soluble mediators, including eicosanoids, growth factors, and adenosine diphosphate (ADP), as well as extracellular vesicles (EVs) rich in microRNAs. Therefore, platelet activation is connected to and controls tissue inflammatory responses (Contursi et al., 2024). One lipid mediator released from platelets upon activation is thromboxane (TX) A_2_, which plays a central role in platelet aggregation and vasoconstriction (Patrono et al., 2005). TXA_2_ enhances platelet recruitment and stabilizes thrombi (Figure 1) (Hamberg et al., 1975). Platelet-derived TXA_2_ drives vascular inflammation by promoting platelet–leukocyte interactions and endothelial activation, contributing to atherosclerosis (Belton et al., 2000; Cheng et al., 2002; Huo and Ley, 2004). In a mouse model of salt-sensitive hypertension [high-salt diet in mice with prostacyclin (PGI_2_) receptor IP deletion (IPKO)], increased TXA_2_ biosynthesis *in vivo* is linked to cardiac fibrosis associated with platelet extravasation and accumulation in the cardiac tissue (D'Agostino et al., 2021). In colorectal adenoma development, enhanced platelet TXA_2_ biosynthesis fosters an inflammatory microenvironment *in vivo*, characterized by cyclooxygenase (COX-2) overexpression and enhanced prostaglandin (PG)E_2_ biosynthesis (Bruno et al., 2022). PGE_2_ is involved in tumorigenesis through multiple mechanisms (apoptosis inhibition, proliferation, migration, angiogenesis, and immune escape) (Wilson and DuBois, 2022) (Figure 1). The role of platelet TXA_2_ in intestinal adenoma development was shown by generating *Apc* ^*Min/+*^ mice with a specific COX-1 deletion in megakaryocytes and platelets. The *Apc* ^*Min/+*^ mouse carries an inactivated allele of the adenomatous polyposis coli (*Apc*) tumor suppressor gene, leading to multiple intestinal adenomas resembling human familial adenomatous polyposis (FAP) (Moser et al., 1995). *Apc* ^*Min/+*^ mice with a specific COX-1 deletion in platelets are characterized by selective inhibition of platelet TXA_2_ biosynthesis associated with fewer intestinal adenomas showing lower COX-2 expression, decreased markers of inflammation and proliferation than *Apc* ^*Min/+*^ mice expressing COX-1 in platelets (Bruno et al., 2022). Several lines of evidence show that activated platelets contribute to tumour metastasis and cancer-associated thrombosis (Gay and Felding-Habermann, 2011). Overall, these lines of evidence underscore the pivotal role of platelet activation and TXA_2_ in the development of cardiovascular disease and cancer, highlighting its shared mechanistic contribution to both conditions and supporting targeting platelet TXA_2_ for therapeutic prevention.

## 2 Biosynthesis of TXA_2_ and insights into the expression, structure, and function of TXA_2_ receptors

**FIGURE 2:**
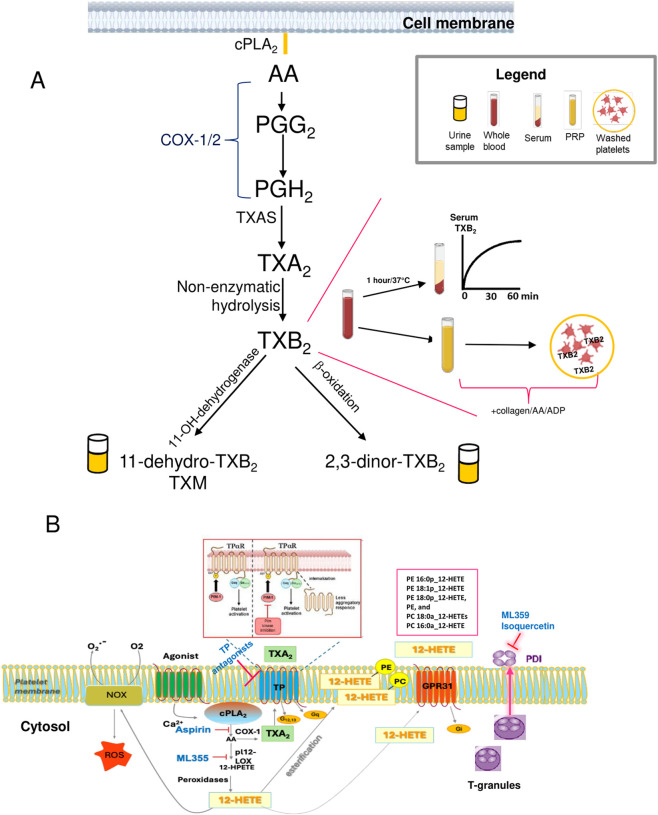
**(A)** TXA_2_ biosynthesis and assessment. The biosynthetic pathway of TXA_2_ involves three enzymatic steps: (i) cytosolic phospholipase A_2_ (cPLA_2_), which contribute to the releases of AA from membrane phospholipids; (ii) the conversion of AA firstly into prostaglandin (PG)G_2_ by the cyclooxygenase (COX) activity of COX-1 and COX-2; then, PGG_2_ is reduced to PGH_2_ by their peroxidase activity; and (iii) TXA_2_ synthase (TXAS), which transforms PGH_2_ to TXA_2_ (Patrignani and Patrono, 2015). COX-1 is constitutively and ubiquitously expressed in the body (Patrignani and Patrono, 2015; Ricciotti and FitzGerald, 2011). It is highly expressed in platelets, and the coordinated activity of COX-1 and TXAS produces large amounts of TXA_2_ (Patrignani and Patrono, 2015; Ricciotti and FitzGerald, 2011). TXA_2_ is chemically unstable with a very short half-life (approximately 30 s), thus it is rapidly hydrolyzed to TXB_2_, which is subjected to dehydrogenation to 11-dehydro-TXB_2_, and to β-oxidation to 2,3-dinor-TXB_2_ by the enzymatic activities in liver, lung, and kidney. TXB_2_ is measured in serum and reflects the maximal biosynthetic capacity of platelets to generate TXA_2_ in response to endogenously formed thrombin during whole-blood clotting after 1 h of incubation at 37 °C. TXB_2_ levels can be measured in plasma-rich platelets (PRP) or in the supernatant of washed platelets after stimulation with different stimuli (AA, collagen, ADP). *In vivo*, systemic TXA_2_ biosynthesis is evaluated by measuring urinary levels of 2,3-dinor-TXB_2_ and 11-dehydro-TXB_2_ (TXM), with TXM being a major enzymatic metabolite. **(B)** Platelet targets as potential candidates for developing safe anti-thrombotic agents: inhibitors of PIM kinase, 12-LOX, and PDI. AA is also metabolized by platelet-type 12-LOX (pl12-LOX) that generates 12-HpETE. (hydroperoxyeicosatetraenoic acid). Cellular peroxidases can reduce HpETE to monohydroxy fatty acids (HETEs). TXA_2_ and 12S-HETE are released and can potentiate platelet activation by interacting with specific G-protein-coupled receptors, TP and GPR31, respectively. A substantial quantity of 12-HETE is esterified into six distinct phospholipid species, consequently modifying platelets to exhibit a procoagulant phenotype. 12S-HETE has been shown to stimulate NADPH oxidases (NOX), and thus enhances the production of ROS (reactive oxygen species), which are known to potentiate platelet activation. Phosphorylation of TPα within the intracellular loop by the serine/threonine-protein kinase pim-1 (PIM1) maintains TPα surface expression and regulates TXA_2_-dependent functional responses. Protein disulfide isomerase (PDI) is localized within platelet T-granules and on the cellular surface. ML-355 inhibits pl12-LOX activity. TXA_2_ biosynthesis is inhibited by aspirin, which affects COX-1 activity; PIM kinase inhibitors reduce the constitutive surface expression of the TXA_2_ receptors TPαRs; ML-359 and isoquercetin inhibit PDI.

TXA_2_ is a lipid mediator of the eicosanoid pathway, generated by COXs from arachidonic acid (AA) (Patrignani and Patrono, 2015) (Figure 2A). Platelets express high levels of COX-1, but not COX-2, and generate PGG_2_ and PGH_2_; PGH_2_ is then transformed to TXA_2_ by the activity of the TXA_2_ synthase (TXAS). Platelets, as well as macrophages, neutrophils, and endothelial cells, synthesize and release TXA_2_ (Smyth, 2010; Fu et al., 1990). Studies in humans treated with low-dose aspirin, which selectively inhibits platelet COX-1, and in mice with specific deletion of COX-1 in platelets show that 75%–80% of *in vivo* TXA_2_ biosynthesis is derived from platelets (Sacco et al., 2019; Bruno et al., 2022). TXA_2_ exerts its biological effects by activating specific TXA_2_ receptors (TP), which are G-protein–coupled receptors (GPCRs). Humans express two TP isoforms, named TPα and TPβ, generated via alternative splicing of a single gene on chromosome 19 (Hirata et al., 1991; Nüsing et al., 1993; Nakahata, 2008). The isoforms share the first 328 amino acids and differ only in their cytoplasmic tails (15 amino acids in TPα, 79 in TPβ). The TP gene (*TBXA2R*) has four exons and three introns; alternative splicing of exon three produces TPα or TPβ depending on the promoter used (Coyle et al., 2002; Coyle and Kinsella, 2005). Promoter (Prm)1 exclusively regulates TPα expression, whereas Prm3 governs TPβ (Coyle et al., 2002; Coyle and Kinsella, 2005; Coyle and Kinsella, 2006; Gannon and Kinsella, 2008; Gannon and Kinsella, 2009). TPα predominates in vascular smooth muscle, endothelial cells, trophoblasts, platelets, and several organs, as well as in cancer cells, whereas TPβ is strongly expressed in endothelial cells (Miggin and Kinsella, 1998). TP receptors form homo- and heterodimers, including with PGI_2_ receptor IP, affecting ligand recognition and signaling (Nakahata, 2008). TP activation regulates cytoskeletal organization, cell adhesion, motility, transcription factor activity, proliferation, survival, and apoptosis via multiple G proteins (G_q_, G_11_, G_12/13_, G_i_, G_s_) and downstream effectors like phospholipase C, RhoGEFs, and adenylyl cyclase. TP signaling is modulated by desensitization, internalization, recycling, oligomerization, and phosphorylation by GPCR kinases, which recruit β-arrestins and clathrin for receptor trafficking (Li et al., 2011). Recently, phosphorylation of TPα at serine 57 within the intracellular loop by the serine/threonine-protein kinase pim-1 (PIM1) has been identified as a novel regulatory mechanism that maintains TPα surface expression and thereby regulates TXA_2_-dependent functional responses (Nock et al., 2025) (Figure 2B).

## 3 Antiplatelet agents targeting the TXA_2_ pathway

### 3.1 COX inhibitors

The most widely used antiplatelet agent targeting the platelet TXA_2_ biosynthesis is aspirin (acetylsalicylic acid, ASA). Aspirin is a nonsteroidal anti-inflammatory drug (NSAID) that, at low doses, exhibits antithrombotic efficacy by irreversibly inhibiting platelet COX-1 activity via acetylation of a serine at position 529 of the COX active site (Roth and Majerus, 1975; Picot et al., 1994). This modification prevents AA from accessing the catalytic active site of the COX channel. This effect results in persistent inhibition of platelet COX-1-dependent TXA_2_ biosynthesis throughout the dosing interval (24 h), despite the drug’s short half-life of 20 min, due to platelets' limited capacity for *de novo* protein synthesis, as they are anucleated cells (Patrono et al., 2001). Low doses (75–100 mg/day) of aspirin preferentially affect platelet COX-1 and result in virtually complete inhibition of platelet TXA_2_ biosynthesis, with limited impact on extraplatelet sources of COX activity (Patrignani et al., 1982).

### 3.2 Thromboxane synthase inhibitors

An alternative strategy to inhibit TXA_2_ biosynthesis involves targeting TXAS, the enzyme that converts PGH_2_ to TXA_2_ (Narumiya et al., 1999) (Figure 2). TXAS inhibitors reduce TXA_2_ biosynthesis while allowing PGH_2_ to be redirected toward other downstream synthases, thereby increasing the production of prostanoids such as PGI_2_, a potent antiaggregatory and vasodilatory agent (Kelton and Blajchman, 1980). However, their clinical development has been limited by incomplete platelet function inhibition and the induction of compensatory mechanisms mediated by other AA metabolites. (Audoly et al., 2000). Ozagrel, approved in Japan for asthma and stroke, significantly reduces TXA_2_ generation (Davì et al., 2012). Meta-analyses indicate that ozagrel improves neurological outcomes in acute ischemic stroke without affecting mortality (Zhang et al., 2012).

### 3.3 TP receptor antagonists

An alternative pharmacological strategy targets the TP receptors to block the biological actions of TXA_2_. TP receptor antagonists inhibit TXA_2_-mediated platelet aggregation, vasoconstriction, and inflammatory signaling, and prevent activation by other agonists, including nonenzymatically generated isoprostanes from AA during oxidative stress (Narumiya et al., 1999; Audoly et al., 2000). Despite the development of numerous TP antagonists, few advanced to late-stage clinical trials due to toxicity or limited efficacy. Ramatroban and seratrodast are approved in Japan for the treatment of allergic rhinitis and asthma (Nakahata, 2008). More recently, the oral TP antagonist ifetroban received FDA Orphan Drug and Rare Pediatric Disease designations for Duchenne cardiomyopathy, based on positive phase II FIGHT Duchenne trial results (NCT03340675). Phase II trials are also evaluating ifetroban in cancer metastasis, cardiovascular disease, and pulmonary arterial hypertension (NCT03694249, NCT03962855, NCT02682511).

### 3.4 Dual-acting agents

To improve efficacy, dual-acting agents that combine TXS inhibition with TP receptor antagonism (Tilley et al., 2001) have been developed, including ridogrel. The rationale is to achieve profound TXA_2_ inhibition, accompanied by increased PGI_2_ production (Xiang et al., 2019). However, the RAPT trial (Ridogrel Versus Aspirin Patency Trial) failed to demonstrate superiority over aspirin in patients with myocardial infarction (RAPT, 1994). Picotamide is licensed in Italy for arterial thrombosis and peripheral artery disease (PAD); it is more effective than aspirin in reducing mortality among type 2 diabetic patients with PAD (Neri Serneri et al., 2004). Recently, a new class of NSAIDs named COXTRAN (COX inhibitor Thromboxane Antagonist) was synthesized with the aim of reducing the cardiovascular toxicity of coxibs, i.e., the selective COX-2 inhibitors (Grosser et al., 2006). These molecules integrate COX-2 inhibition with TP receptor antagonism. One compound, upon oral administration in a murine model, reduced inflammatory pain through COX-2 inhibition and concurrently inhibited TXA_2_-dependent platelet aggregation by preventing platelet TP activation, thereby offering the potential to mitigate the possible increased thrombotic risk associated with COX-2 inhibitors. (Blua et al., 2025; Grosser et al., 2006).

### 3.5 PIM kinase inhibitors

The deletion of the Provirus Integration site for the Moloney murine leukemia virus (Pim) gene, along with the use of pharmacological inhibitors, has been shown to decrease thrombus formation by reducing platelet responses and decreasing TPαR surface expression, without affecting primary hemostasis (Unsworth et al., 2021). Thus, PIM kinase inhibitors have the potential to develop safer drugs to prevent cardiovascular disease (Patrignani et al., 2025). Nock et al. (2025) reported that PIM kinase-1-dependent phosphorylation of serine 57 (S57) in the TPα receptors’ first intracellular loop is involved in the constitutive surface expression of TPα receptors (Figure 2B). The potential of these inhibitors to induce antitumor effects by reducing TPα surface expression across various cell types warrants a comprehensive investigation. Notably, the investigational oral PIM1 kinase-selective inhibitor, nuvisertib (TS3654), has been granted Fast Track Designation by the Food and Drug Administration (FDA) for the treatment of immediate or high-risk myelofibrosis (Dutta et al., 2022).

## 4 Assessment of TXA_2_ biosynthesis

TXA_2_ is a chemically unstable autacoid with a short half-life; it is converted to the stable, nonenzymatic hydration product TXB_2_, which is rapidly metabolized enzymatically, resulting in very low circulating levels. Plasma TXB_2_ measurements are not advised because of artefactual platelet activation and TXB_2_ release during blood sampling (FitzGerald et al., 1983). When precautions are taken and a high-sensitivity assay is used, plasma TXB_2_ is undetectable in healthy individuals (FitzGerald et al., 1983). However, high levels of TXB_2_ are generated in whole blood allowed to clot for 1 h at 37 °C (serum TXB_2_) (Patrono et al., 1980). Serum TXB_2_ serves as a biomarker of platelet COX-1 activity *ex vivo*, reflecting the maximum capacity of platelets to produce TXA_2_ in response to endogenously formed thrombin. This measurement is widely used to assess pharmacological inhibition of platelet COX-1 in health and disease (Patrono and Rocca, 2019). TXB_2_ levels can also be measured in platelet-rich plasma, or washed platelet supernatants after stimulation with AA, collagen, or ADP (Tacconelli et al., 2020).

*In vivo*, systemic biosynthesis of TXA_2_/TXB_2_ is assessed by measuring urinary 2,3-dinor-TXB_2_ and 11-dehydro-TXB_2_ (TXM) (Figure 2A), their main enzymatic urinary metabolites formed via hepatic beta-oxidation and 11-OH-dehydrogenation, respectively (Patrono and Rocca, 2019). Assessment of urinary TXM levels indicates the TXA_2_/TXB_2_ entry rate into circulation and offers a time-integrated measure of *in vivo* TXA_2_ biosynthesis. Low-dose aspirin (100 mg/day) reduces urinary TXM levels by approximately 70%–80% in healthy subjects, reflecting the platelet contribution to TXA_2_ generation *in vivo* (Reilly and FitzGerald, 1987; Patrignani et al., 2014); thus, its increase implicates platelet activation. Elevated urinary TXM levels have been measured in association with cardiovascular risk factors, i.e., hypertension and renovascular hypertension (Minuz et al., 2002), diabetes mellitus (Petrucci et al., 2024), and hypercholesterolemia (Davì et al., 1992). Systemic TXA_2_ generation, assessed by measuring urinary TXM, independently predicts cardiovascular events and mortality in both aspirin users and non-users, regardless of traditional risk factors (Eikelboom et al., 2008; Rade et al., 2022). In addition, in patients with colorectal cancer (CRC) and Familial Adenomatous Polyposis (FAP), baseline urinary TXM levels exceed the upper cutoff values for healthy individuals (Patrignani et al., 2014; 2017; Lanas et al., 2023; Sciulli et al., 2005). A substudy of the ongoing Add-Aspirin trial (ISRCTN74358648) investigated the impact of two aspirin doses (100 and 300 mg/day) after radical cancer therapy across four tumor types, including CRC. The study found that urinary TXM remained elevated, particularly in CRC and gastro-oesophageal cancer (Joharatnam-Hogan et al., 2023). Findings showed that both 100 mg and 300 mg/day of aspirin reduced urinary TXM levels equally, consistent with the saturation of platelet COX-1 inhibition at low doses (Joharatnam-Hogan et al., 2023). Further research is needed to determine whether monitoring urinary TXM could serve as an early marker for tumor development or recurrence, especially in gastrointestinal cancers.

## 5 Anticancer effects of aspirin

### 5.1 Results of clinical studies

Extensive clinical research indicates that aspirin offers protective benefits against cancer, especially CRC (Patrignani and Patrono, 2016; Patrono et al., 2026). Regular use of low-dose aspirin is associated with a decreased risk of CRC and other gastrointestinal malignancies, as demonstrated by meta-analyses of randomized clinical trials aimed at cardiovascular prevention (Rothwell et al., 2010; 2012). This protective effect seems to be dose-independent across the range of 75–300 mg/day, indicating that platelet inhibition by aspirin, rather than direct anticancer activity in tumor tissue, is likely involved.

Lynch syndrome is a hereditary condition that markedly increases the risk of various cancers, particularly colorectal and endometrial cancers, often before age 50. It results from mutations in DNA repair genes such as *MLH1*, *MSH2*, *MSH6*, and *PMS2* (Eroglu et al., 2025). The CaPP2 trial (Burn et al., 2020) involving Lynch syndrome patients demonstrated that aspirin (300 mg twice daily) significantly reduces CRC incidence, with benefits observed years after drug cessation, suggesting a role for aspirin in inhibiting early tumorigenic mechanisms. Consequently, the National Institute for Health and Care Excellence (NICE) currently recommends that individuals with Lynch syndrome consider daily aspirin therapy (Recommendation 1.1.1 of the NICE colorectal cancer guideline), though specific dosing guidance has not been issued. The CaPP3 trial was therefore designed to identify the optimal aspirin dose for cancer prevention while minimizing side effects in Lynch syndrome carriers. This non-inferiority trial compares aspirin 100 mg/day with 300 and 600 mg/day. Follow-up of eligible gene carriers has now exceeded 5 years, and preliminary data support the non-inferiority of the 100 mg dose compared to higher doses (Patrono et al., 2026). Based on results from the CaPP2 and CaPP3 trials, it is recommended that Lynch syndrome patients take 75–100 mg of aspirin daily to reduce cancer risk.

Adjuvant treatment with 160 mg aspirin daily for 3 years to reduce recurrence rate in CRC patients with somatic alterations in the PI3K signaling pathway was assessed in the ALASCCA (Adjuvant Low-Dose Aspirin in Colorectal Cancer) trial (Martling et al., 2025). It was a randomized, double-blind, multicenter study conducted across 33 hospitals in Sweden, Denmark, Finland, and Norway. It involved patients with stage I-III rectal or stage II-III colon cancer exhibiting PI3K pathway alterations. The primary endpoint was CRC recurrence in patients with specific *PIK3CA* mutations in exons nine or 20. The 3-year cumulative incidence of recurrence was 7.7% with aspirin and 14.1% with placebo among these patients (HR 0.49; 95% CI 0.24–0.98). For patients with other PI3K pathway alterations, recurrence was 7.7% with aspirin and 16.8% with placebo (HR 0.42; 95% CI 0.21). The mechanism by which aspirin exerts anticancer effects in patients with PI3K mutations still needs to be clarified. Data from over 7,000 participants in various randomized clinical trials (some ongoing and some concluded) evaluating aspirin as an adjuvant therapy for CRC will be analyzed in the PROSPERO-registered Prospective Aspirin Meta-analysis (CRD42023453156). This meta-analysis aims to evaluate the benefits and risks of aspirin in the management of CRC. While participants in most trials were enrolled regardless of molecular subtype, some trials include secondary analyses focusing on tumors with *PIK3CA* mutations. If this study confirms aspirin’s anticancer effects in patients with *PIK3CA* mutations, these findings could lead to personalized aspirin therapy in cancer treatment, potentially changing clinical practice for about one-third of CRC patients with PI3K mutations (Goldberg and Meyerhardt, 2025; Drew et al., 2025).

### 5.2 Aspirin prevents metastasis by limiting platelet TXA_2_ suppression of T cell immunity in mice

Multiple lines of evidence indicate that platelets play a crucial role in regulating tumor metastasis (Gay and Felding-Habermann, 2011). Recent research demonstrates that platelet-derived TXA_2_ acts as an immunosuppressive mediator, inhibiting T cell activity and facilitating metastatic growth (Figure 1). Yang et al. (2025) reported that inhibition of platelet COX-1, either pharmacologically with aspirin or through platelet-specific COX-1 deletion, markedly enhances immune-mediated control of metastasis in murine models. Mechanistically, TXA_2_ directly activates an immunosuppressive signaling pathway in T cells that relies on the guanine exchange factor ARHGEF1. This activation results in the suppression of T cell receptor (TCR)–driven kinase signaling, proliferation, and effector functions (Yang et al., 2025) (Figure 1). Consistent with these findings, conditional deletion of *Arhgef1* in T cells increases activation at metastatic sites and promotes immune-mediated rejection of lung and liver metastases. Collectively, these results reveal a platelet-driven immunosuppressive pathway involving TXA_2_/TP/ARGEF1 that constrains T cell immunity during dissemination. This mechanistic insight offers a compelling explanation for the anti-metastatic effects of aspirin and supports the advancement of novel immune-based strategies targeting the platelet COX-1/TXA_2_ axis to prevent cancer metastasis. These results are promising and shed light on how aspirin prevents metastasis; however, they are preclinical evidence, and it is important to validate this pathway in humans.

## 6 Ongoing development of novel antiplatelet agents to minimize bleeding side effects

Novel antiplatelet agents targeting traditional platelet pathways have been discussed in previous reviews (Majithia and Bhatt, 2019; Gawaz et al., 2023). The development of innovative therapies that target additional receptor and signaling pathways—focusing on maintaining antiplatelet effects while protecting hemostasis—has great potential to improve clinical outcomes for patients with atherothrombotic diseases and cancer (Majithia and Bhatt, 2019; Gawaz et al., 2023). In this review, we report some promising options, including protein disulfide isomerase (PDI) inhibitors, 12-lipoxygenase (LOX) inhibitors, and targeting the PIM kinase pathway (which we described above) (see Figure 2B).

### 6.1 PDI inhibitors

PDI is a thiol isomerase that modifies disulfide bonds during protein synthesis and folding (Flaumenhaft et al., 2015). It functions as either a reductase or oxidase, influenced by factors such as redox environment, pH, allosteric modulators, substrate properties, and localization (Ellgaard and Ruddock, 2005). Mainly located in the endoplasmic reticulum (ER) of nucleated cells, PDI is also found in platelets within T-granules (Thon et al., 2012). During activation, PDI is released from platelet and endothelial cell granules, playing a key role in thrombus formation (Essex and Wang, 2024). It is also expressed on the cell surface of nucleated cells and platelets (Cho et al., 2012; Flaumenhaft et al., 2015). It may be retained by a KDEL sequence that facilitates surface expression (Bartels et al., 2019). Extracellular PDI interacts with platelet receptor αIIbβ3, promoting platelet activation (Wang et al., 2019; Wang et al., 2023). PDIA1 supports platelet activation by regulating the Nox1-ROS-TXA_2_ pathway, which is crucial for collagen-induced activation of αIIbβ3 (Przyborowski et al., 2022). Modulating platelet PDI is a promising approach for developing antiplatelet therapies by targeting Nox1, which is associated with reducing bleeding risk (Vara et al., 2021). PDI inhibitors are a significant focus of ongoing research, particularly in the contexts of cancer, cardiovascular diseases, and neurodegenerative conditions. While numerous compounds show promise in preclinical evaluations, no PDI-specific drug has yet received regulatory approval for clinical use. Isoquercetin (also known as quercetin-3-O-beta-D-glucoside or isoquercitrin) and quercetin-3-rutinoside (rutin) (Zwicker et al., 2019) are currently undergoing clinical trials aimed at preventing venous thromboembolism, especially among cancer patients. ML359 is a second-generation PDI inhibitor that selectively blocks PDI oxidoreductase activity and has enhanced properties compared to the first-generation PDI inhibitors (Flaumenhaft et al., 2015).

### 6.2 12-LOX inhibitors

Platelet-type 12-LOX promotes platelet activation and metastasis. It metabolizes AA to produce 12S-HpETE (hydroperoxyeicosatetraenoic acid), which is subsequently converted into 12S-HETE (hydroxyeicosatetraenoic acid). The latter can activate the G protein-coupled receptor GPR31, which couples to G_i_/G_o_ proteins (Guo et al., 2011). Notably, 12S-HETE produced by platelets is incorporated into membrane phospholipids such as phosphatidylcholine and phosphatidylethanolamine, thereby contributing to thrombin formation (Thomas et al., 2010). 12-LOX also regulates platelet signaling via FcγRIIA, a receptor that binds immune complexes to trigger platelet activation (Patel et al., 2021; Yeung et al., 2014; Luci et al., 2013). ML355 is a selective small-molecule inhibitor of 12-LOX with nanomolar potency (Luci et al., 2013). In experimental animals, the compound demonstrated antithrombotic effects with minimal impact on primary hemostasis (Adili et al., 2017). There is a potential for the development of 12-LOX inhibitors as antitumor agents, capable of preventing cancer cell-derived IgG-dependent platelet activation and cancer-associated thrombosis.

## 7 Conclusion

The role of platelets in the development and progression of cancer, particularly CRC, is increasingly acknowledged, with platelet-derived TXA_2_ playing a significant role. TXA_2_ influences cancer through two main mechanisms: firstly, by promoting inflammation at gastrointestinal mucosal lesions via COX-2 induction and increased PGE_2_ production; secondly, by suppressing T-cell-mediated anti-cancer immune responses (Patrono et al., 2026; Langley and Burn, 2025). Low-dose aspirin (75–100 mg/day) acts as a selective and irreversible inhibitor of platelet-derived TXA_2_ by targeting platelet COX-1 (Patrono et al., 2001). Recent results from the large randomized clinical trial CaPP3 in patients with Lynch syndrome demonstrate that low-dose aspirin is as effective as higher doses (300 mg/day and 600 mg/day) in reducing bowel cancer incidence. These findings suggest that the anticancer effects of aspirin in Lynch syndrome primarily result from its inhibition of platelet function. Furthermore, the data support the use of a lower aspirin dose as a safer long-term preventative measure, moving away from the previously tested 600 mg/day dose in the CaPP2 study (Burn et al., 2020). Additionally, findings from the ALASCCA trial (Martling et al., 2025) indicate that low-dose aspirin (160 mg/day) significantly decreases the risk of cancer recurrence in patients with *PIK3CA* hotspot mutations in exons nine or 20 and appears to offer similar benefits among those with other somatic alterations in PI3K pathway genes. The mechanisms underlying the response to low-dose aspirin in *PIK3CA* mutation carriers remain to be elucidated, with ongoing research in numerous laboratories. The potential role of aspirin as an adjunctive therapy for CRC in patients with or without alterations in PI3K pathway genes is to be confirmed through ongoing clinical trials and a comprehensive meta-analysis registered with PROSPERO (CRD42023453156) (Drew et al., 2025). It is important to note that chronic use of low-dose aspirin may increase bleeding risk, particularly in older adults (Patrono et al., 2026). Its application in primary and secondary prevention should be guided by personalized strategies incorporating biomarkers for early detection, disease monitoring, and risk assessment. Urinary TXM levels show promise as indicators of platelet activation and may help identify individuals at increased risk of CRC. Further research is necessary to explore the potential benefits of other conventional antiplatelet agents, TP receptor antagonists, and novel therapeutic agents under clinical development that modulate platelet function without impairing primary hemostasis.

## References

[B1] Adili R. Tourdot B. E. Mast K. Yeung J. Freedman J. C. Green A. (2017). First selective 12-LOX inhibitor, ML355, impairs thrombus Formation and vessel occlusion *in vivo* with minimal effects on hemostasis. Arteriosclerosis, Thrombosis, Vasc. Biol. 37 (10), 1828–1839. 10.1161/ATVBAHA.117.309868 28775075 PMC5620123

[B2] Audoly L. P. Rocca B. Fabre J. E. Koller B. H. Thomas D. Loeb A. L. (2000). Cardiovascular responses to the isoprostanes are mediated *via* the thromboxane A_2_ receptor *in vivo* . Circulation 101 (24), 2833–2840. 10.1161/01.cir.101.24.2833 10859290

[B3] Bartels A. K. Göttert S. Desel C. Schäfer M. Krossa S. Scheidig A. J. (2019). KDEL receptor 1 contributes to cell Surface Association of protein disulfide isomerases. Cell. Physiology Biochem. 52 (4), 850–868. 10.33594/000000059 30958660

[B4] Belton O. Byrne D. Kearney D. Leahy A. Fitzgerald D. J. (2000). Cyclooxygenase-1 and -2-dependent prostacyclin and thromboxane formation in human atherosclerosis. Circulation 102 (8), 840–845. 10.1161/01.cir.102.8.840 10952950

[B5] Blua F. Boccato F. Buccellati C. Risè P. Barbieri S. Castiglioni L. (2025). Exploiting the 2-(1,3,4,9-tetrahydropyrano[3,4-b]indol-1-yl)acetic acid scaffold to generate COXTRANs: a new class of dual cyclooxygenase inhibitors-thromboxane receptor antagonists. J. Med. Chem. 68 (21), 23185–23219. 10.1021/acs.jmedchem.5c02068 41124679 PMC12621201

[B6] Bruno A. Contursi A. Tacconelli S. Sacco A. Hofling U. Mucci M. (2022). The specific deletion of cyclooxygenase-1 in megakaryocytes/platelets reduces intestinal polyposis in ApcMin/+ mice. Pharmacol. Res. 185, 106506. 10.1016/j.phrs.2022.106506 36241001

[B7] Burn J. Sheth H. Elliott F. Reed L. Macrae F. Mecklin J. P. (2020). Cancer prevention with aspirin in hereditary colorectal cancer (Lynch syndrome), 10-year follow-up and registry-based 20-year data in the CAPP2 study: a double-blind, randomised, placebo-controlled trial. Lancet 395, 1855–1863. 10.1016/S0140-6736(20)30366-4 32534647 PMC7294238

[B8] Cheng Y. Austin S. C. Rocca B. Koller B. H. Coffman T. M. Grosser T. (2002). Role of prostacyclin in the cardiovascular response to thromboxane A2. Science 296 (5567), 539–541. 10.1126/science.1068713 11964481

[B9] Cho J. Kennedy D. R. Lin L. Huang M. Merrill-Skoloff G. Furie B. C. (2012). Protein disulfide isomerase capture during thrombus formation *in vivo* depends on the presence of β3 integrins. Blood 120 (3), 647–655. 10.1182/blood-2011-08-372532 22653978 PMC3401216

[B10] Contursi A. Tacconelli S. Di Berardino S. De Michele A. Patrignani P. (2024). Platelets as crucial players in the dynamic interplay of inflammation, immunity, and cancer: unveiling new strategies for cancer prevention. Front. Pharmacol. 15, 1520488. 10.3389/fphar.2024.1520488 39764464 PMC11701038

[B11] Coyle A. T. Kinsella B. T. (2005). Characterization of promoter 3 of the human thromboxane A receptor gene. A functional AP-1 and octamer motif are required for basal promoter activity. FEBS J. 272 (4), 1036–1053. 10.1111/j.1742-4658.2004.04538.x 15691336

[B12] Coyle A. T. Kinsella B. T. (2006). Synthetic peroxisome proliferator-activated receptor gamma agonists rosiglitazone and troglitazone suppress transcription by promoter 3 of the human thromboxane A2 receptor gene in human erythroleukemia cells. Biochem. Pharmacol. 71 (9), 1308–1323. 10.1016/j.bcp.2006.01.011 16499875

[B13] Coyle A. T. Miggin S. M. Kinsella B. T. (2002). Characterization of the 5’ untranslated region of alpha and beta isoforms of the human thromboxane A2 receptor (TP). Differential promoter utilization by the TP isoforms. Eur. J. Biochem. 269 (16), 4058–4073. 10.1046/j.1432-1033.2002.03102.x 12180983

[B14] D'Agostino I. Tacconelli S. Bruno A. Contursi A. Mucci L. Hu X. (2021). Low-dose Aspirin prevents hypertension and cardiac fibrosis when thromboxane A2 is unrestrained. Pharmacol. Res. 170, 105744. 10.1016/j.phrs.2021.105744 34182131

[B15] Davì G. Averna M. Catalano I. Barbagallo C. Ganci A. Notarbartolo A. (1992). Increased thromboxane biosynthesis in type IIa hypercholesterolemia. Circulation 85 (5), 1792–1798. 10.1161/01.cir.85.5.1792 1572035

[B16] Davì G. Santilli F. Vazzana N. (2012). Thromboxane receptors antagonists and/or synthase inhibitors. Handb. Exp. Pharmacol. (210), 261–286. 10.1007/978-3-642-23056-1_10 22918735

[B17] De La Cruz A. Hargrave A. Magadi S. Courson J. A. Landry P. T. Zhang W. (2021). Platelet and erythrocyte extravasation across inflamed corneal venules depend on CD18, neutrophils, and mast cell degranulation. Int. J. Mol. Sci. 22 (14), 7360. 10.3390/ijms22147360 34298979 PMC8329926

[B18] Drew D. A. Downie J. M. Chan A. T. (2025). Piking the right patients for adjuvant aspirin therapy for colorectal cancer. Clin. Cancer Res. 31, 3107–3109. 10.1158/1078-0432.CCR-25-0953 40439583

[B19] Dutta A. Nath D. Yang Y. Le B. T. Rahman M. F. Faughnan P. (2022). Genetic ablation of Pim1 or pharmacologic inhibition with TP-3654 ameliorates myelofibrosis in murine models. Leukemia 36 (3), 746–759. 10.1038/s41375-021-01464-2 34741118 PMC8891046

[B20] Eikelboom J. W. Hankey G. J. Thom J. Bhatt D. L. Steg P. G. Montalescot G. (2008). Incomplete inhibition of thromboxane biosynthesis by acetylsalicylic acid: determinants and effect on cardiovascular risk. Circulation 118 (17), 1705–1712. 10.1161/CIRCULATIONAHA.108.768283 18838564

[B21] Ellgaard L. Ruddock L. W. (2005). The human protein disulphide isomerase family: substrate interactions and functional properties. EMBO Rep. 6 (1), 28–32. 10.1038/sj.embor.7400311 15643448 PMC1299221

[B22] Eroglu S. Birsenogul I. Bowen A. P. Doyle J. F. Pupikin S. E. Villar J. (2025). Lynch Syndrome in focus: a multidisciplinary review of cancer risk, clinical management, and special populations. Cancers (Basel) 17 (24), 3981. 10.3390/cancers17243981 41463230 PMC12730836

[B23] Essex D. W. Wang L. (2024). Recent advances in vascular thiol isomerases and redox systems in platelet function and thrombosis. J. Thrombosis Haemostasis 22 (7), 1806–1818. 10.1016/j.jtha.2024.03.008 38518897 PMC11214884

[B24] FitzGerald G. A. Oates J. A. Hawiger J. Maas R. L. Roberts L. J. Lawson J. A. (1983). Endogenous biosynthesis of prostacyclin and thromboxane and platelet function during chronic administration of aspirin in man. J. Clin. Investigation 71 (3), 676–688. 10.1172/JCI110814 6338043 PMC436917

[B25] Flaumenhaft R. Furie B. Zwicker J. I. (2015). Therapeutic implications of protein disulfide isomerase inhibition in thrombotic disease. Arteriosclerosis, Thrombosis, Vasc. Biol. 35 (1), 16–23. 10.1161/ATVBAHA.114.303410 25104801 PMC4270882

[B26] Fu J. Y. Masferrer J. L. Seibert K. Raz A. Needleman P. (1990). The induction and suppression of prostaglandin H2 synthase (cyclooxygenase) in human monocytes. J. Biol. Chem. 265 (28), 16737–16740. 10.1016/S0021-9258(17)44818-4 2120205

[B27] Gannon A. M. Kinsella B. T. (2008). Regulation of the human thromboxane A2 receptor gene by Sp1, Egr1, NF-E2, GATA-1, and Ets-1 in megakaryocytes. J. Lipid Res. 49 (12), 2590–2604. 10.1194/jlr.M800293-JLR200 18698092

[B28] Gannon A. M. Kinsella B. T. (2009). The Wilms’ tumour suppressor protein WT1 acts as a key transcriptional repressor of the human thromboxane A2 receptor gene in megakaryocytes. J. Cell. Mol. Med. 13 (11–12), 4571–4586. 10.1111/j.1582-4934.2008.00511.x 19067769 PMC4515072

[B29] Gawaz M. Geisler T. Borst O. (2023). Current concepts and novel targets for antiplatelet therapy. Nat. Rev. Cardiol. 20, 583–599. 10.1038/s41569-023-00851-x 37016032

[B30] Gay L. J. Felding-Habermann B. (2011). Contribution of platelets to tumour metastasis. Nat. Rev. Cancer 11 (2), 123–134. 10.1038/nrc3004 21258396 PMC6894505

[B31] Goldberg R. M. Meyerhardt J. A. (2025). An aspirin a day for improved colorectal cancer outcomes. N. Engl. J. Med. 393, 1131–1132. 10.1056/NEJMe2508634 40961430

[B32] Grosser T. Fries S. FitzGerald G. A. (2006). Biological basis for the cardiovascular consequences of COX-2 inhibition: therapeutic challenges and opportunities. J. Clin. Investigation 116 (1), 4–15. 10.1172/JCI27291 16395396 PMC1323269

[B33] Guo Y. Zhang W. Giroux C. Cai Y. Ekambaram P. Dilly A. K. (2011). Identification of the orphan G protein-coupled receptor GPR31 as a receptor for 12-(S)-hydroxyeicosatetraenoic acid. J. Biol. Chem. 286 (39), 33832–33840. 10.1074/jbc.M111.233486 21712392 PMC3190773

[B34] Hamberg M. Svensson J. Samuelsson B. (1975). Thromboxanes: a new group of biologically active compounds. Proc. Natl. Acad. Sci. U. S. A. 72 (8), 2994–2998. 10.1073/pnas.72.8.2994 1059088 PMC432905

[B35] Harrison P. (2005). Platelet function analysis. Blood Rev. 19 (2), 111–123. 10.1016/j.blre.2004.05.002 15603914

[B36] Hirata M. Hayashi Y. Ushikubi F. Yokota Y. Kageyama R. Nakanishi S. (1991). Cloning and expression of cDNA for a human thromboxane A2 receptor. Nature 349 (6310), 617–620. 10.1038/349617a0 1825698

[B37] Holinstat M. (2017). Normal platelet function. Cancer Metastasis Rev. 36 (2), 195–198. 10.1007/s10555-017-9677-x 28667366 PMC5709181

[B38] Huo Y. Ley K. F. (2004). Role of platelets in the development of atherosclerosis. Trends Cardiovasc Med. 14 (1), 18–22. 10.1016/j.tcm.2003.09.007 14720470

[B39] Joharatnam-Hogan N. Hatem D. Cafferty F. H. Petrucci G. Cameron D. A. Ring A. (2023). Thromboxane biosynthesis in cancer patients and its inhibition by aspirin: a sub-study of the Add-Aspirin trial. Br. J. Cancer 129, 706–720. 10.1038/s41416-023-02310-1 37420000 PMC10421951

[B40] Kelton J. G. Blajchman M. A. (1980). Prostaglandin I2 (prostacyclin). Can. Med. Assoc. J. 122, 175–179. 6988063 PMC1801769

[B41] Lanas A. Tacconelli S. Contursi A. Piazuelo E. Bruno A. Ronci M. (2023). Biomarkers of response to low-dose aspirin in familial adenomatous polyposis patients. Cancers (Basel) 15, 2457. 10.3390/cancers15092457 37173923 PMC10177499

[B42] Langley R. E. Burn J. (2025). Understanding how aspirin prevents metastasis. N. Engl. J. Med. 393, 2368–2371. 10.1056/NEJMcibr2502386 41370804

[B95] Lecomte M. Laneuville O. Ji C. DeWitt D. L. Smith W. L. (1994). Acetylation of human prostaglandin endoperoxide synthase-2 (cyclooxygenase-2) by aspirin. J. Biol. Chem. 269, 13207–13215. 8175750

[B99] Lefrançais E. Ortiz-Muñoz G. Caudrillier A. Mallavia B. Liu F. Sayah D. M. (2017). The lung is a site of platelet biogenesis and a reservoir for haematopoietic progenitors. Nature 544 (7648), 105–109. 10.1038/nature21706 28329764 PMC5663284

[B96] Li D. D’Angelo L. Chavez M. Woulfe D. S. (2011). Arrestin-2 differentially regulates PAR4 and ADP receptor signaling in platelets. J. Biol. Chem. 286 (5), 3805–3814. 10.1074/jbc.M110.169110 21106537 PMC3030382

[B43] Luci D. Jameson J. B. I. I. Yasgar A. Diaz G. Joshi N. Kantz N. (2013). Discovery of ML355, a potent and selective inhibitor of human 12-Lipoxygenase. Bethesda (MD): National Center for Biotechnology Information US. 25506969

[B44] Majithia A. Bhatt D. L. (2019). Novel antiplatelet therapies for atherothrombotic diseases. Arterioscler. Thromb. Vasc. Biol. 39 (4), 546–557. 10.1161/ATVBAHA.118.310955 30760019 PMC6445601

[B45] Martling A. Hed Myrberg I. Nilbert M. Grönberg H. Granath F. Eklund M. (2025). Low-Dose aspirin for PI3K-Altered localized colorectal cancer. N. Engl. J. Med. 393 (11), 1051–1064. 10.1056/NEJMoa2504650 40961426

[B46] Michelson A. D. (2002). Platelets. San Diego: Academic Press, 3–19.

[B47] Miggin S. M. Kinsella B. T. (1998). Expression and tissue distribution of the mRNAs encoding the human thromboxane A2 receptor (TP) alpha and beta isoforms. Biochim. Biophys. Acta 1425 (3), 543–559. 10.1016/s0304-4165(98)00109-3 9838218

[B48] Minuz P. Patrignani P. Gaino S. Degan M. Menapace L. Tommasoli R. (2002). Increased oxidative stress and platelet activation in patients with hypertension and renovascular disease. Circulation 106 (22), 2800–2805. 10.1161/01.cir.0000039528.49161.e9 12451006

[B49] Moser A. R. Luongo C. Gould K. A. McNeley M. K. Shoemaker A. R. Dove W. F. (1995). ApcMin: a mouse model for intestinal and mammary tumorigenesis. Eur. J. Cancer 31A (7-8), 1061–1064. 10.1016/0959-8049(95)00181-h 7576992

[B50] Nakahata N. (2008). Thromboxane A2: physiology/pathophysiology, cellular signal transduction and pharmacology. Pharmacol. & Ther. 118 (1), 18–35. 10.1016/j.pharmthera.2008.01.001 18374420

[B51] Narumiya S. Sugimoto Y. Ushikubi F. (1999). Prostanoid receptors: structures, properties, and functions. Physiol. Rev. 79 (4), 1193–1226. 10.1152/physrev.1999.79.4.1193 10508233

[B52] Neri Serneri G. G. Coccheri S. Marubini E. Violi F. (2004). Picotamide, a combined inhibitor of thromboxane A2 synthase and receptor, reduces 2-year mortality in diabetics with peripheral arterial disease: the DAVID study. Eur. Heart J. 25 (20), 1845–1852. 10.1016/j.ehj.2004.07.013 15474700

[B53] Nie D. Lamberti M. Zacharek A. Li L. Szekeres K. Tang K. (2000). Thromboxane A(2) regulation of endothelial cell migration, angiogenesis, and tumor metastasis. Biochem. Biophysical Res. Commun. 267 (1), 245–251. 10.1006/bbrc.1999.1840 10623605

[B54] Nock S. Hutchinson J. L. Blanco-Lopez M. Naseem K. Jones S. Mundell S. J. (2025). Constitutive surface expression of the Thromboxane A2 receptor is Pim kinase dependent. J. Thrombosis Haemostasis 23 (2), 293–305. 10.1016/j.jtha.2024.09.013 39798965

[B55] Nüsing R. M. Hirata M. Kakizuka A. Eki T. Ozawa K. Narumiya S. (1993). Characterization and chromosomal mapping of the human thromboxane A2 receptor gene. J. Biol. Chem. 268 (34), 25253–25259. 10.1016/S0021-9258(19)74595-5 8227091

[B56] Patel P. Michael J. V. Naik U. P. McKenzie S. E. (2021). Platelet FcγRIIA in immunity and thrombosis: adaptive immunothrombosis. J. Thrombosis Haemostasis 19 (5), 1149–1160. 10.1111/jth.15265 33587783

[B57] Patrignani P. Patrono C. (2015). Cyclooxygenase inhibitors: from pharmacology to clinical read-outs. Biochimica Biophysica Acta (BBA) - Mol. Cell Biol. Lipids 1851 (4), 422–432. 10.1016/j.bbalip.2014.09.016 25263946

[B58] Patrignani P. Patrono C. (2016). Aspirin and cancer. J. Am. Coll. Cardiol. 68 (9), 967–976. 10.1016/j.jacc.2016.05.083 27561771

[B59] Patrignani P. Filabozzi P. Patrono C. (1982). Selective cumulative inhibition of platelet thromboxane production by low-dose aspirin in healthy subjects. J. Clin. Invest 69 (6), 1366–1372. 10.1172/jci110576 7045161 PMC370209

[B60] Patrignani P. Tacconelli S. Piazuelo E. Di Francesco L. Dovizio M. Sostres C. (2014). Reappraisal of the clinical pharmacology of low-dose aspirin by comparing novel direct and traditional indirect biomarkers of drug action. J. Thrombosis Haemostasis 12 (8), 1320–1330. 10.1111/jth.12631 24942808

[B61] Patrignani P. Sacco A. Sostres C. Bruno A. Dovizio M. Piazuelo E. (2017). Low-Dose aspirin acetylates Cyclooxygenase-1 in Human Colorectal mucosa: implications for the chemoprevention of Colorectal cancer. Clin. Pharmacol. & Ther. 102 (1), 52–61. 10.1002/cpt.639 28139830

[B62] Patrignani P. De Michele A. Tacconelli S. (2025). New antiplatelet approach: inhibiting Pim kinase to reduce constitutive surface expression of thromboxane A2 receptor. J. Thrombosis Haemostasis 23 (1), 43–46. 10.1016/j.jtha.2024.10.027 39798970

[B63] Patrono C. Rocca B. (2019). Measurement of Thromboxane biosynthesis in health and disease. Front. Pharmacol. 10, 1244. 10.3389/fphar.2019.01244 31736753 PMC6832017

[B64] Patrono C. Ciabattoni G. Pinca E. Pugliese F. Castrucci G. De Salvo A. (1980). Low dose aspirin and inhibition of thromboxane B2 production in healthy subjects. Thrombosis Res. 17 (3-4), 317–327. 10.1016/0049-3848(80)90066-3 7368167

[B65] Patrono C. Coller B. Dalen J. E. FitzGerald G. A. Fuster V. Gent M. (2001). Platelet-active drugs: the relationships among dose, effectiveness, and side effects. Chest 119 (1 Suppl. l), 39S–63S. 10.1378/chest.119.1_suppl.39s 11157642

[B66] Patrono C. García Rodríguez L. A. Landolfi R. Baigent C. (2005). Low-dose aspirin for the prevention of atherothrombosis. N. Engl. J. Med. 353 (22), 2373–2383. 10.1056/NEJMra052717 16319386

[B67] Patrono C. Burn J. Patrignani P. Langley R. E. (2026). Platelet activation, aspirin, and cancer: from basic science to clinical trials. Pharmacol. Rev. 78 (1), 100109. 10.1016/j.pharmr.2025.100109 41592353 PMC7619208

[B68] Petrucci G. Buck G. A. Rocca B. Parish S. Baigent C. Hatem D. (2024). Thromboxane biosynthesis and future events in diabetes: the ASCEND trial. Eur. Heart J. 45 (15), 1355–1367. 10.1093/eurheartj/ehad868 38385506 PMC11015956

[B69] Picot D. Loll P. J. Garavito R. M. (1994). The X-ray crystal structure of the membrane protein prostaglandin H2 synthase-1. Nature 367 (6460), 243–249. 10.1038/367243a0 8121489

[B70] Przyborowski K. Kurpinska A. Wojkowska D. Kaczara P. Suraj-Prazmowska J. Karolczak K. (2022). Protein disulfide isomerase-A1 regulates intraplatelet reactive oxygen species-thromboxane A2-dependent pathway in human platelets. J. Thrombosis Haemostasis 20 (1), 157–169. 10.1111/jth.15539 34592041 PMC9292974

[B71] Rade J. J. Barton B. A. Vasan R. S. Kronsberg S. S. Xanthakis V. Keaney J. F. (2022). Association of thromboxane generation with survival in aspirin users and nonusers. J. Am. Coll. Cardiol. 80 (3), 233–250. 10.1016/j.jacc.2022.04.034 35660296 PMC12175083

[B72] RAPT (1994). Randomized trial of ridogrel, a combined thromboxane A2 synthase inhibitor and thromboxane A2/prostaglandin endoperoxide receptor antagonist, *versus* aspirin as adjunct to thrombolysis in patients with acute myocardial infarction. The Ridogrel *versus* Aspirin Patency Trial (RAPT). Circulation 89 (2), 588–595. 10.1161/01.cir.89.2.588 8313547

[B73] Reilly I. A. FitzGerald G. A. (1987). Inhibition of thromboxane formation *in vivo* and *ex vivo*: implications for therapy with platelet inhibitory drugs. Blood 69 (1), 180–186. 3790723

[B74] Ricciotti E. FitzGerald G. A. (2011). Prostaglandins and inflammation. Arteriosclerosis, Thrombosis, Vasc. Biol. 31 (5), 986–1000. 10.1161/ATVBAHA.110.207449 21508345 PMC3081099

[B75] Rodgers G. M. (1999). Overview of platelet physiology and laboratory evaluation of platelet function. Clin. Obstetrics Gynecol. 42 (2), 349–359. 10.1097/00003081-199906000-00014 10370853

[B76] Roth G. J. Majerus P. W. (1975). The mechanism of the effect of aspirin on human platelets. I. Acetylation of a particulate fraction protein. J. Clin. Investigation 56 (3), 624–632. 10.1172/JCI108132 1159076 PMC301910

[B77] Rothwell P. M. Wilson M. Elwin C. E. Norrving B. Algra A. Warlow C. P. (2010). Long-term effect of aspirin on colorectal cancer incidence and mortality: 20-year follow-up of five randomised trials. Lancet 376 (9754), 1741–1750. 10.1016/S0140-6736(10)61543-7 20970847

[B78] Rothwell P. M. Price J. F. Fowkes F. G. Zanchetti A. Roncaglioni M. C. Tognoni G. (2012). Short-term effects of daily aspirin on cancer incidence, mortality, and non-vascular death: analysis of the time course of risks and benefits in 51 randomised controlled trials. Lancet 379 (9826), 1602–1612. 10.1016/S0140-6736(11)61720-0 22440946

[B79] Sacco A. Bruno A. Contursi A. Dovizio M. Tacconelli S. Ricciotti E. (2019). Platelet-Specific deletion of Cyclooxygenase-1 ameliorates Dextran Sulfate Sodium-Induced colitis in mice. J. Pharmacol. Exp. Ther. 370 (3), 416–426. 10.1124/jpet.119.259382 31248980

[B80] Sciulli M. G. Filabozzi P. Tacconelli S. Padovano R. Ricciotti E. Capone M. L. (2005). Platelet activation in patients with colorectal cancer. Prostagl. Leukot. Essent. Fat. Acids 72 (2), 79–83. 10.1016/j.plefa.2004.10.010 15626589

[B81] Smyth E. M. (2010). Thromboxane and the thromboxane receptor in cardiovascular disease. Clin. Lipidol. 5 (2), 209–219. 10.2217/clp.10.11 20543887 PMC2882156

[B82] Tacconelli S. Fullone R. Dovizio M. Pizzicoli G. Marschler S. Bruno A. (2020). Pharmacological characterization of the biosynthesis of prostanoids and hydroxyeicosatetraenoic acids in human whole blood and platelets by targeted chiral lipidomics analysis. Biochimica Biophysica Acta (BBA) - Mol. Cell Biol. Lipids 1865 (12), 158804. 10.1016/j.bbalip.2020.158804 32853794

[B83] Thomas C. P. Morgan L. T. Maskrey B. H. Murphy R. C. Kühn H. Hazen S. L. (2010). Phospholipid-esterified eicosanoids are generated in agonist-activated human platelets and enhance tissue factor-dependent thrombin generation. J. Biol. Chem. 285 (9), 6891–6903. 10.1074/jbc.M109.078428 20061396 PMC2844139

[B84] Thon J. N. Peters C. G. Machlus K. R. Aslam R. Rowley J. Macleod H. (2012). T granules in human platelets function in TLR9 organization and signaling. J. Cell Biol. 198 (4), 561–574. 10.1083/jcb.201111136 22908309 PMC3514030

[B85] Tilley S. L. Coffman T. M. Koller B. H. (2001). Mixed thromboxane synthase inhibition and TP receptor antagonism in thrombosis and inflammation. Trends Pharmacol. Sci. 22 (6), 306–312. 10.1016/S0165-6147(00)01710-5 11395159

[B86] Unsworth A. J. Bye A. P. Sage T. Gaspar R. S. Eaton N. Drew C. (2021). Antiplatelet properties of Pim kinase inhibition are mediated through disruption of thromboxane A2 receptor signaling. Haematologica 106 (7), 1968–1978. 10.3324/haematol.2019.223529 32467143 PMC8252961

[B87] Vara D. Mailer R. K. Tarafdar A. Wolska N. Heestermans M. Konrath S. (2021). NADPH oxidases are required for full platelet activation *in vitro* and thrombosis *in vivo* but dispensable for plasma coagulation and hemostasis. Arterioscler. Thromb. Vasc. Biol. 41, 683–697. 10.1161/ATVBAHA.120.315565 33267663 PMC7837688

[B88] Wang L. Zhou J. Wang L. Wang C. C. Essex D. W. (2019). The b' domain of protein disulfide isomerase cooperates with the a and a' domains to functionally interact with platelets. J. Thromb. Haemost. 17, 371–382. 10.1111/jth.14366 30566278 PMC6368866

[B97] Wang L. Wang J. Li J. Walz T Coller B. S. (2023). Studies of protein disulfide isomerase (PDI) binding to platelets and production of a murine monoclonal antibody (mAb) to integrin αIIbβ3 that inhibits PDI binding and platelet aggregation. Blood 142 (1), 1200. 10.1182/blood-2023-174416

[B89] Wilson D. J. DuBois R. N. (2022). Role of prostaglandin E2 in the progression of gastrointestinal cancer. Cancer Prev. Res. (Phila). 15, 355–363. 10.1158/1940-6207.CAPR-22-0038 35288737 PMC9359060

[B90] Xiang Q. Pang X. Liu Z. Yang G. Tao W. Pei Q. (2019). Progress in the development of antiplatelet agents: focus on the targeted molecular pathway from bench to clinic. Pharmacol. Ther. 203, 107393. 10.1016/j.pharmthera.2019.107393 31356909

[B91] Yang J. Yamashita-Kanemaru Y. Morris B. I. Contursi A. Trajkovski D. Xu J. (2025). Aspirin prevents metastasis by limiting platelet TXA2 suppression of T cell immunity. Nature 640, 1052–1061. 10.1038/s41586-025-08626-7 40044852 PMC12018268

[B92] Yeung J. Tourdot B. E. Fernandez-Perez P. Vesci J. Ren J. Smyrniotis C. J. (2014). Platelet 12-LOX is essential for FcγRIIa-mediated platelet activation. Blood 124, 2271–2279. 10.1182/blood-2014-05-575878 25100742 PMC4183986

[B93] Zhang J. Yang J. Chang X. Zhang C. Zhou H. Liu M. (2012). Ozagrel for acute ischemic stroke: a meta-analysis of data from randomized controlled trials. Neurol. Res. 34, 346–353. 10.1179/1743132812Y.0000000022 22643078

[B98] Zwicker J. I. Schlechter B. L. Stopa J. D. Liebman H. A Aggarwal A. Puligandla M. (2019). Targeting protein disulfide isomerase with the flavonoid isoquercetin to improve hypercoagulability in advanced cancer. JCI Insight 4 (4), e125851. 10.1172/jci.insight.125851 30652973 PMC6478409

